# Insights into RNA N6-methyladenosine and programmed cell death in atherosclerosis

**DOI:** 10.1186/s10020-024-00901-z

**Published:** 2024-09-03

**Authors:** Haijiao Long, Yulu Yu, Jie Ouyang, Hongwei lu, Guojun Zhao

**Affiliations:** 1 Health Management Center, The Third Xiangya Hospital of Central South University, Changsha, 410013 China; 2 Department of Cardiology, The Third Xiangya Hospital of Central South University, Changsha, 410013 China; 3 Afliated Qingyuan Hospital, Guangzhou Medical University (Qingyuan People’s Hospital), Qingyuan, Guangdong China

**Keywords:** Atherosclerosis, N6-methyladenosine, Programmed cell death, Diagnosis, Treatment

## Abstract

N6-methyladenosine (m^6^A) modification stands out among various RNA modifications as the predominant form within eukaryotic cells, influencing numerous cellular processes implicated in disease development. m^6^A modification has gained increasing attention in the development of atherosclerosis and has become a research hotspot in recent years. Programmed cell death (PCD), encompassing apoptosis, autophagy, pyroptosis, ferroptosis, and necroptosis, plays a pivotal role in atherosclerosis pathogenesis. In this review, we delve into the intricate interplay between m^6^A modification and diverse PCD pathways, shedding light on their complex association during the onset and progression of atherosclerosis. Clarifying the relationship between m^6^A and PCD in atherosclerosis is of great significance to provide novel strategies for cardiovascular disease treatment.

## Introduction

N6-methyladenosine (m^6^A) (see Glossary), a methylation modification of the sixth adenine (A) nitrogen, is one of the most common markers of post-transcriptional regulation that occurs in different types of RNAs, particularly in eukaryotic mRNAs (Niu et al. 2013). Recent studies have shown that m^6^A plays a catalytic role in the development of atherosclerosis, which is the cause of a majority of cardiovascular events. For example, m^6^A methyltransferase-like protein 14 (METTL14) can promote atherosclerosis via endothelial and macrophage inflammation, respectively (Dong et al. 2021; Jian et al. 2020; Zheng et al. 2022).

Programmed cell death (PCD), which includes classical apoptosis, autophagy, pyroptosis, ferroptosis and necroptosis, etc. is a subset of regulated cell death. PCD responds to internal or external threats in a positively regulated manner and is essential for maintaining normal cell cycle and tissue homeostasis (Kist and Vucic 2021; Bedoui et al. 2020). Mounting evidence has been accumulating that PCD plays a fundamental role in the development of atherosclerosis by lysing and effectively eliminating inflammatory cells (Paone et al. 2019; Pi et al. 2021; Wu et al. 2018).

As the most common epigenetic modification on RNA, m^6^A methylation affects RNA metabolism and thus controls PCD progression in a post-transcriptional manner. In this review, we focus on the current associations between m^6^A and different types of PCD pathways in the occurrence and development of atherosclerosis. We also highlight the potential diagnostic and therapeutic value of the m^6^A and PCD pathways in atherosclerosis.

## Molecular compositions of m^6^A methylation

**Fig. 1:**
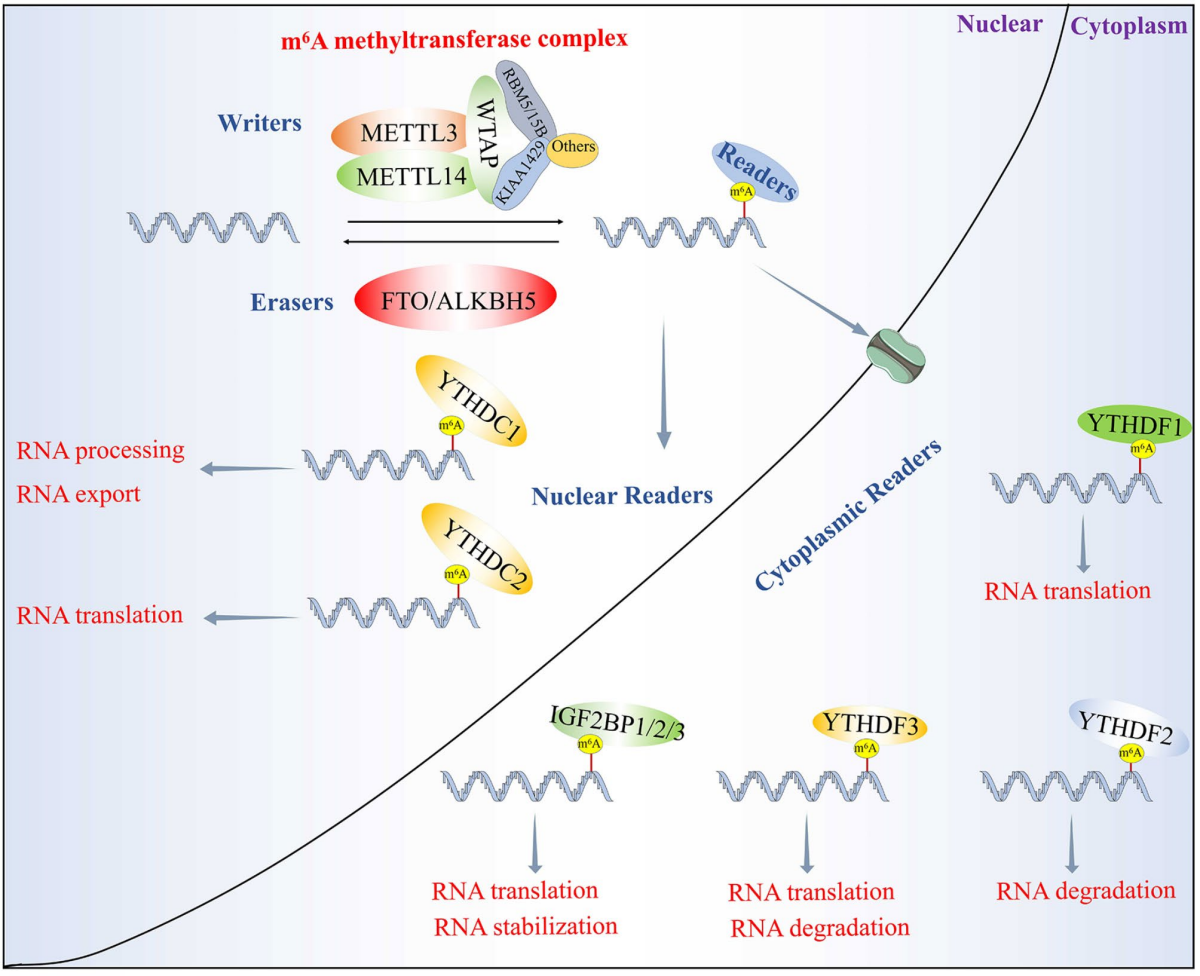
m^6^A modification regulators. m^6^A is incorporated into mRNAs in the nucleus by the methyltransferase complex, which includes proteins such as METTL3, METTL14, WTAP, RBM5/15, KIAA1429, etc. The methyl group can be removed by demethylases, including FTO and ALKBH5. RNA incorporating m^6^A can be recognized by readers such as YTHDC1, YTHDC2, YTHDF1, YTHDF2, YTHDF3 and IGF2BP1/2/3. m^6^A is involved in almost the entire process on mRNA, including splicing, translation, export, stabilisation, degradation. etc

The m^6^A methyltransferase (writers), binding protein (readers) and demethylase (erasers) are involved in the initiation, recognition and removal of RNAs, respectively (Fig. 1). m^6^A modification is regulated by the m^6^A methylase complex, which consists of METTL3, METTL14, Wilms tumor-associated protein (WTAP), RNA-binding motif protein 15 (RBM15), etc. (Zaccara et al. 2019; Shi et al. 2019). Among them, METTL3 and METTL14 are the major catalytic cores, and their methyltransferase domains have catalytic activity (Liu et al. 2014; Wang et al. 2016). WTAP stabilizes the METTL3-METTL14 complex and promotes the RNA-binding ability of m^6^A methyltransferase (Ping et al. 2014; Schöller et al. 2018). RBM15 can interact with METTL3 in a WTAP-dependent manner to recruit the complex to methylate specific sites (Patil et al. 2016). However, the molecular function of other m^6^A writers in the m^6^A methylase complex remains unknown, such as Vir like m^6^A methyltransferase associated (VIRMA, also known as KIAA1429).

In response to external forces, m^6^A modification is selectively recognized by several RNA-binding proteins (RBPs), triggering a series of downstream biological responses. YTH domain containing 1 (YTHDC1) bind m^6^A-modified mRNAs, and promotes RNA processing and export (Roundtree et al. 2017; Chen et al. 2021; Liang et al. 2022). Meanwhile, YTHDC2 can improve the incidence of translation of target mRNA by binding m^6^A-modified RNAs (Ma et al. 2021; Yuan et al. 2022). The YTH domain family protein family (YTHDF) is also m^6^A readers, which includes three paralogs, YTHDF1, YTHDF2, and YTHDF3. YTHDF1 enhances mRNA translation (Yuan et al. 2022), YTHDF2 promotes mRNA degradation (Li et al. 2020; Hou et al. 2021), and YTHDF3 enhances both translation and degradation (Liao et al. 2022; Chang et al. 2020). Similarly, insulin-like growth factor 2 mRNA binding proteins (IGF2BP1/2/3) promote m^6^A-modified mRNA stabilization and protein translation (Huang et al. 2018; Sun et al. 2022; He et al. 2022). As a dynamic reversible RNA modification, m^6^A can be demethylated by erasers. Fat mass and obesity-associated protein (FTO) and alkB homolog 5 (ALKBH5) have been identified as m^6^A erasers (Xu et al. 2020; Chen et al. 2019; Mathiyalagan et al. 2019). It should be noted that these m^6^A regulators are not independent components, they all maintain the balance of m^6^A methylation of RNA in a sophisticated manner.

## Function of m^6^A methylation on RNA metabolism

m^6^A methylation is involved in mRNA metabolism, which includes mRNA splicing, translation, nuclear export, decay, etc. (Roundtree et al. 2017; Zhou et al. 2019; Wu et al. 2021; Zhang et al. 2022a, b, c) In mRNA sequences, transcripts carry m^6^A modifications mainly around 3’-UTR or 5’-UTR, and coding sequences (CDS) (Berulava et al. 2020). Of these, approximately 41.9% were distributed in the UTRs and 50.9% in the coding region (Meyer et al. 2012). Different locations of m^6^A methylation sites can lead to complex effects on the mRNA. In particular, most transcripts with methylation in the 3’-UTR are associated with metabolic processes, whereas transcripts containing m^6^A in the 5’-UTR or CDS are associated with energy metabolism, mitochondrial function, and intracellular pathways (Berulava et al. 2020; Kasowitz et al. 2018; Zhou et al. 2015). However, the detailed mechanism still needs to be further investigated.

In recent years, studies have shown that m^6^A modification is also an indispensable part of the processing of non-coding RNAs (ncRNAs), which are divided into three categories, long non-coding RNAs (lncRNAs), microRNAs (miRNAs), and circular RNAs (circRNAs) (Slack and Chinnaiyan 2019). Interestingly, not only m^6^A methylation regulate various aspects of ncRNAs biology, but ncRNAs also regulate the level of m^6^A methylation modification by altering the m^6^A modification regulators. For example, the lncRNA SNHG4 decreases the m^6^A level of STAT2 mRNA by inhibiting METTL3 expression (Li et al. 2021).

## m^6^A modification and PCD in atherosclerosis

M^6^A modification was first identified in apoptosis, and then more and more studies have demonstrated that m^6^A regulators could interact with almost all types of PCD pathways. Here, we mainly discuss the relationship between m^6^A and cell apoptosis, autophagy, pyroptosis, and necroptosis in the occurrence and development of atherosclerosis (Table 1).

**Table 1:** m^6^A modification and PCD in atherosclerosis

Regulator	Expression	Types of PCD	Molecular mechanisms	Model	Ref
METTL3	Increased	Apoptosis	Increased NPC1L1 expression	HUVECs and ApoE^−/−^ mice	(Zhang et al. 2023a)
METTL3/ALKBH5 /YTHDF3	Increased METTL3 or decreased ALKBH5	Apoptosis	Increased MCU mRNA translation	Vascular endothelial cells	(Zhu et al. 2022)
METTL14	Increased	Apoptosis	Increased p65 mRNA stability	HUVECs and ApoE^−/−^ mice	(Liu et al. 2022)
ALKBH5	Decreased	Apoptosis	Decreased Bcl-2 expression and increased miR-7	HUVECs	(Zhang et al. 2022b
FTO	Increased	Autophagy	Increased ULK1 protein level	HeLa-GFP-LC3B cells	(Jin et al. 2018)
YTHDF2	Increased	Autophagy	Increased ULK1 mRNA degradation	HeLa-GFP-LC3B cells	(Jin et al. 2018)
METTL3	Increased	Autophagy	Increased ATG5 and ATG7 expression	HASMCs	(Fang et al. 2023)
METTL3	Increased	Pyroptosis	Increased MALAT1 level via m^6^A methylation, which promoted USP8 mRNA degradation through the interaction with PTBP1	Pro-inflammatory M1 macrophages	(Shu et al. 2021)
METTL3	Increased	Pyroptosis	Decreased circ_0029589 through promoting its m^6^A modification	Human and macrophage	(Guo et al. 2020)
YTHDC1	Increased	Pyroptosis	m^6^A-induced decay of FENDRR promotes HPAEC pyroptosis by regulating DRP1 promoter methylation	HPAECs	(Wang et al. 2022)
METTL3	Increased	Ferroptosis	Increased GPX4 level via m^6^A modification	Alveolar epithelial cells	(Zhang et al. 2022c)
METTL3	Increased	Ferroptosis	Decreased SLC7A11 and FSP1 expression	SMCs	(Li et al. 2022)
WTAP	Increased	Necroptosis	m^6^A modification destabilizes Orai1 and Ripk1 mRNAs	T cell	(Ito-Kureha et al. 2022)
ALKBH5	Decreased	Necroptosis	Nuclear HIF-1α bound to the ALKBH1-demethylated MIAT promoter and transcriptionally upregulated its expression	human endothelium and monocyte cells/leukocytes and endothelium in western diet-induced AS mice	(Wu et al. 2019)
IGF2BP3	Increased	Necroptosis	Increased HIF1A expression	MKN-45 cells and HUVECs	(Jiang et al. 2021)

### Apoptosis

**Fig. 2:**
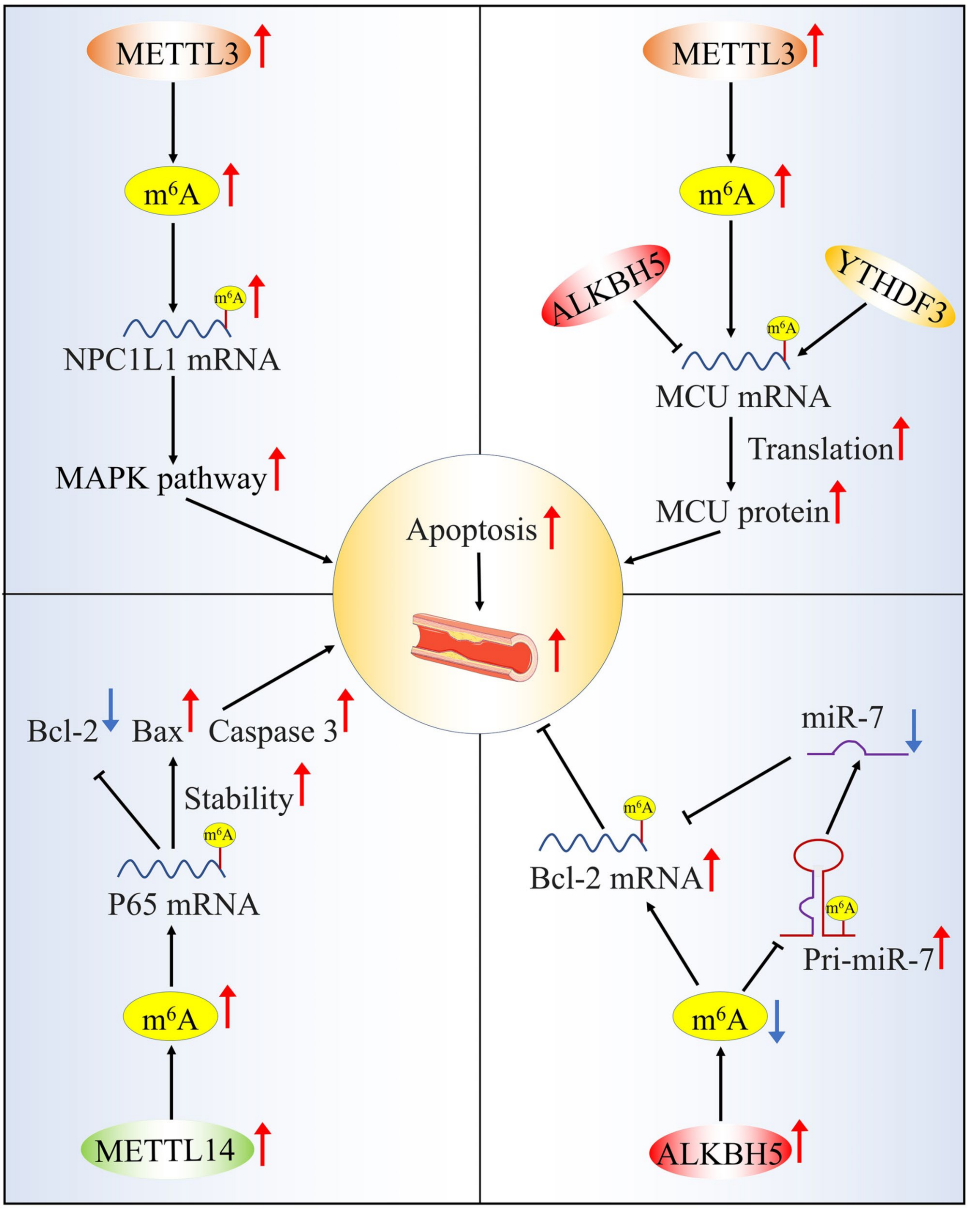
m^6^A modification and apoptosis in atherosclerosis. METTL3-mediated NPC1L1 mRNA hypermethylation facilitates atherosclerosis progression by regulating the MAPK pathway. METTL3 and YTHDF3-mediated m^6^A methylation induces vascular endothelial cell apoptosis by promote increased MCU translation; ALKBH5 reverses METTL3-mediated m^6^A modification of MCU mRNA; METTL14 is involved in endothelial cell apoptosis and plaque development by regulating m^6^A modification of p65; ALKBH5 promoted the expression of Bcl-2 in HUVEC apoptosis; Meanwhile, ALKBH5 regulates Bcl-2 expression by decreasing the level of miR-7

Apoptosis occurs throughout all stages of atherosclerosis. METTL3 gene mediated Niemann-Pick C1-like protein 1 (NPC1L1) mRNA hypermethylation facilitates atherosclerosis progression through endothelial cell apoptosis (Zhang et al. 2023a, b). Zhu et al. found that METTL3 is also the major contributor to HCMV-induced apoptosis of vascular endothelial cells (Zhu et al. 2022). Mechanically, METTL3 promotes the incorporation of YTHDF3 into methylated calcium uniporter (MCU) mRNA, then increases MCU translation and expression, and thereby enhancing HCMV-induced apoptosis of vascular endothelial cells. Further analysis showed that ALKBH5 was the demethylase of MCU mRNA, which could negatively regulate the m^6^A modification process of MCU. Reduction of METTL14 has been reported to reduce ox-LDL-induced endothelial cell apoptosis and aortic plaque area in *ApoE*^*−/−*^ mice treated with a high-fat diet (Liu et al. 2022). Epigenetically, METTL14-mediated m^6^A methylation regulated the protein expressions of bax and cleaved caspase-3 through influences p65 stability and its expression. In addition to the writer protein, the eraser protein also plays an important role. The results observed by Zhang et al. indicated that ALKBH5 overexpression inhibited the apoptosis in TNF-α-treated HUVECs by promoting the expression of Bcl-2 (Zhang et al. 2022a, b, c). Meanwhile, ALKBH5 overexpression also increased the level of pri-miR-7 and decreased the level of miR-7. MiR-7 was reported to significantly inhibit the expression of Bcl-2 (Zhu et al. 2019). These results expand the understanding of the progression mechanism of atherosclerosis and provide a potential strategy for the protection of vascular endothelial injury (Fig. 2). However, m^6^A-mediated apoptosis of other atherosclerotic cells, such as macrophages and vascular smooth muscle cells (VSMCs), may have additional effects that remain to be discovered .

### Autophagy

**Fig. 3:**
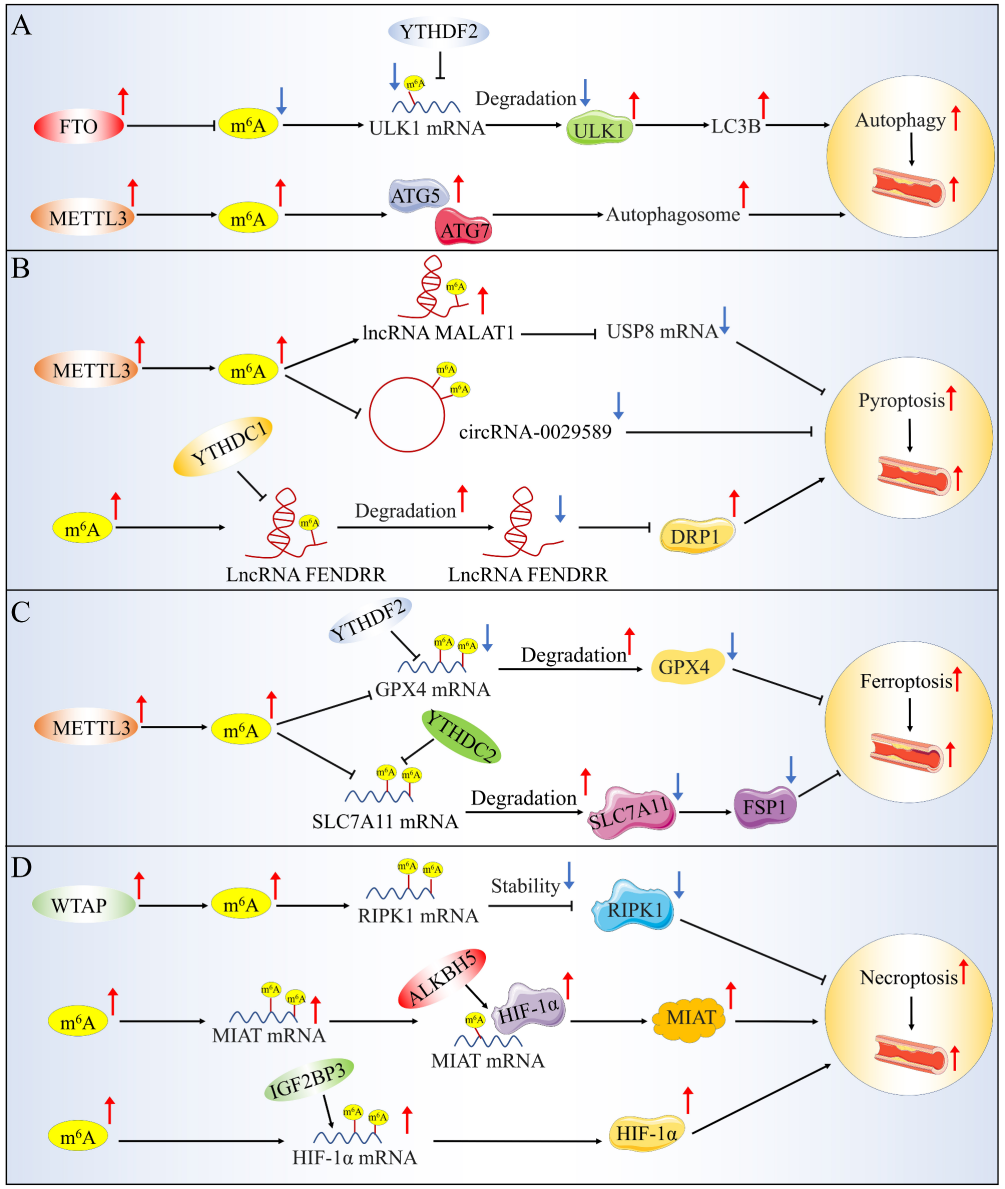
m^6^A modification and autophagy, pyroptosis, ferroptosis and necroptosis in atherosclerosis. (**A**) m^6^A modification and autophagy. FTO eliminates the m^6^A modification of ULK1, reduces its degradation by YTHDF2 and promotes the production of LC3BII; METTL3 promotes autophagosome formation by upregulating ATG5 and ATG7 expression. (**B**) m^6^A modification and pyroptosis. METTL3 promotes macrophage pyroptosis by increasing lncRNA MALAT1 m^6^A methylation and decreasing hsa_circ_0029589 expression; YTHDC1 regulates the methylation of DRP1 promoter by inducing the degradation of LncRNA FENDRR, which further promotes endothelial cell pyroptosis. (**C**) m^6^A modification and ferroptosis. METTL3-YTHDF2 promoted alveolar epithelial cell ferroptosis through mediated m^6^A modification of GPX4; METTL3-SLC7A11/FSP1 axis regulates HASMC ferroptosis. (**D**) m^6^A modification and necroptosis. WTAP regulates T-cell necroptosis by disrupting the stability of RIPK1 mRNA; ALKBH5 promotes atherosclerosis by regulating the binding of HIF-1α to MIAT in human endothelium and monocytes; IGF2BP3 regulates HIF-1α expression by directly binding to a specific m^6^A site

Autophagy is an essential PCD mechanism through frequent changes in autophagy-related proteins and transcription factors (Mizushima et al. 2011). Autophagy plays an important role in the degradation of proteins and damaged organelles, and autophagy dysfunction in vascular cells is closely associated with atherosclerosis (Grootaert et al. 2018; Sergin and Razani 2014). Since the relationship between autophagy and m^6^A was first studied in 2018, its role in the occurrence and development of atherosclerosis has been further explored (Fig. 3A). Jin et al. performed a screen by using small interfering RNAs targeting genes encoding the writers, erasers, and readers proteins and identified the FTO protein as a positive regulator of autophagy (Jin et al. 2018). FTO can directly abrogate the m^6^A distribution in Unc-51 like autophagy activating kinase (ULK1), thereby reducing its degradation by YTHDF2 and further promoting the production of ULK1 protein and LC3BII (Jin et al. 2018). ULK1 plays a central role in the initiation of autophagy, a process that contributes to atherosclerosis (Ouyang et al. 2021a, b; Luo et al. 2017). m^6^A has also been implicated as an autophagy activator in the study of VSMCs. Enhanced METTL3 promotes autophagosome formation by upregulating the expression of autophagy-related proteins 5 and 7 (ATG5 or ATG7), and further inhibits VSMC migration and synthetic phenotype formation (Fang et al. 2023). In addition, knockout of ATG5 or ATG7 largely reversed the regulatory effect of METTL3 overexpression on the phenotypic transformation of HASMCs, as manifested by increased proliferation and migration and a preference for synthetic phenotypes (Fang et al. 2023). The proliferation of VSMCs may be beneficial throughout atherogenesis, and not only in advanced lesions (Bennett et al. 2016). The experimental results that both writer protein METTL3 and the eraser protein FTO promote autophagy are apparently contradictory. The reason may be that overexpression of FTO eliminated m^6^A levels in ULK1 transcripts and simultaneously reduced YTHDF2-mediated mRNA decay. These results suggest that the m^6^A modifier acts as a double-edged sword in autophagy, which is likely to depend on abundant RNA-binding proteins and their recognition locations. However, despite the growing recognition of the biological significance of m^6^A modification, the overall influence of m^6^A regulators on the transcription and translation of other autophagy-related genes in the development of atherosclerosis remains poorly understood. Meanwhile, whether autophagy can regulate m^6^A components is also worthy of further exploration.

### Pyroptosis

Pyroptosis is a pro-inflammatory form of regulated cell death characterized by cell swelling, the protrusion of large bubbles from the plasma membrane and cell lysis (Xu et al. 2018). This death pathway is mediated by gasdermin D (GSDMD) pore formation, which is activated by caspase-1, and followed by the release of both cell contents and pro-inflammatory cytokines, which play a prominent role in the development of atherosclerosis, particularly in unstable atherosclerotic lesions (He et al. 2021). The involvement of METTL3-mediated m^6^A modification in the pyroptosis of pro-inflammatory macrophages has recently been reported. Upregulation of METTL3 increases lncRNA MALAT1 levels through m^6^A methylation, promotes degradation of ubiquitin specific peptidase 8 (USP8) mRNA, and then promotes pyroptosis and inflammation of macrophages (Shu et al. 2021). IFN regulatory factor (IRF)-1 is considered to be a potent activator of macrophage pyroptosis and inflammation (Dai et al. 2022). The latest studies have elucidated that IRF-1 inhibits the expression of hsa_circ_0029589 by inducing m^6^A levels and METTL3 expression, thereby promoting macrophages pyroptosis and inflammatory response in atherosclerosis (Guo et al. 2020). Meanwhile, the m^6^A reader YTHDC1 has also been reported to play an important role in the degradation of m^6^A-modified LncRNA FENDRR (Wang et al. 2022). m^6^A-induced LncRNA FENDRR degradation promotes endothelial cells pyroptosis by regulating the methylation of the dynamin-related protein 1 (DRP1) promoter (Wang et al. 2022). In conclusion, these implications crucially build a bridge between m^6^A and pyroptosis-related components in the development of atherosclerosis (Fig. 3B). However, more research is warranted to explore other RNAs with m^6^A modifications in pyroptosis.

### Ferroptosis

Ferroptosis is an iron-dependent form of necrosis characterized by oxidative damage to phospholipids, which causes damage to vascular endothelial cells, macrophages and VSMCs etc. and affects many risk factors or pathological processes of atherosclerosis (Ouyang et al. 2021a, b). Recent studies have revealed the association between m^6^A and ferroptosis (Fig. 3C). Neutrophil extracellular traps (NETs) have been shown to promote atherosclerotic plaque formation through a pro-inflammatory immune response (Döring et al. 2017). In sepsis, NETs promote ferroptosis through METTL3-induced m^6^A modification of GPX4 (Zhang et al. 2022a, b, c). In contrast, METTL3 knockout inhibited NETs-induced cell ferroptosis and protected mice against sepsis-associated acute lung injury (Zhang et al. 2022a, b, c). Additionally, METTL3 overexpression has also been shown to promote ferroptosis in HASMCs by inhibiting the expression of key ferroptosis regulatory proteins, such as solute carrier family 7 member 11 (SLC7A11) and ferroptosis suppressor protein 1 (FSP1) (Li et al. 2022). Overexpression of SLC7A11 or FSP1 largely rescues the effect of METTL3 on ferroptosis in HASMC (Li et al. 2022). These studies have linked m^6^A to ferroptosis and indirectly support the role of METTL3 modification-induced ferroptosis in the development of atherosclerosis. Targeting METTL3 can be used as a potential motivator of ferroptosis in atherosclerotic patients to guide clinical treatment.

### Necroptosis

Necroptosis, a novel pro-inflammatory programmed cell death pathway characterised by early loss of cytoplasmic membrane integrity, leakage of cell contents, and organelles swelling, has been implicated in atherosclerosis (Coornaert et al. 2018; Gao et al. 2022). Two kinase proteins that play pivotal roles in the necroptosis pathway, receptor-interacting protein kinase 1 (RIPK1) and RIPK3, have emerged as the key proteins for their destructive role in atherosclerosis (DeRoo et al. 2020). Recent studies have shown that WTAP, one of the key proteins in the function of the m^6^A methyltransferase complex, destabilises RIPK1 mRNA and further regulates T-cell necroptosis (Ito-Kureha et al. 2022). These findings uncover a potential pathway by which m^6^A modifications affect cell necroptosis. Hypoxia-inducible factor (HIF)-1α has been shown to promote macrophage necroptosis by regulating miR-210 and miR-383 (Karshovska et al. 2020). Wu et al. demonstrated that ox-LDL-induced ALKBH5 promoted HIF-1α binding and activating myocardial infarction-associated transcript (MIAT) in human endothelium and monocytes, which further promoted atherosclerosis progression (Wu et al. 2019). Further studies revealed that the m^6^A reader insulin-like growth factor-II mRNA-binding protein 3 (IGF2BP3) positively regulated HIF-1α expression by directly binding to a specific m^6^A site in the coding region of HIF-1α mRNA (Jiang et al. 2021). Taken together, these results support the possibility that m^6^A modifications are actively involved in necroptosis in the development of atherosclerosis (Fig. 3D). However, direct evidence for m^6^A-mediated necroptosis needs to be further investigated.

## Clinical application of the target-based m^6^A-PCD axis in atherosclerosis

Emerging data indicate that the abnormal global abundance of m^6^A, abnormal gene expression levels of m^6^A writers, erasers and readers, and m^6^A site mutations are common in various diseases, providing promising biomarkers for clinical diagnosis and treatment. m^6^A modification-related proteins have multiple functions in PCD, and elucidating their interaction in the pathological process is helpful in discovering specific potential biomarkers and therapeutic targets, providing a strong basis for the diagnosis and treatment of atherosclerosis. In particular, by focusing on the association of m^6^A with apoptosis, autophagy, ferroptosis and necroptosis of vascular cells in atherosclerosis, we cannot ignore the potential value of m^6^A in anti-atherosclerosis. In conclusion, the m^6^A-mediated PCD axis has become an important mechanism in the pathological process. Meanwhile, RNA contains multiple m^6^A sites in the PCD pathway, and targeting multiple m^6^A sites shows more diagnostic and therapeutic potential than targeting a single m^6^A.

### Diagnostic potential

Zhou et al. identified the pathological model of human coronary artery smooth muscle cells (HCASMCs) induced in vitro by RNA-Seq and meRIP-Seq, and identified 5121 m^6^A peaks and 883 mRNAs differentially expressed in the pathological process of HCASMCs (Zhou et al. 2022a, b). Among them, METTL3 is up-regulated in atherosclerotic lesions and HCASMCs proliferation and migration models, and METTL3 knockdown can inhibit the pathological process of HCASMCs. Meanwhile, the expression of peripheral blood mononuclear cell-derived macrophage RNA in patients with coronary artery disease was detected by RNA-Seq, and the expression level of METTL3 was significantly increased. Upregulation of METTL3 can significantly increase the expression of hsa_circ_0029589, and promote macrophage pyroptosis and inflammatory response in atherosclerosis (Guo et al. 2020). These findings may contribute to the expansion of METTL3 as a potential diagnostic marker for atherosclerosis (Table 2). However, other m^6^A-modified proteins are currently lacking in animal and clinical trials, and their diagnostic effect on atherosclerosis is still not perfect, which deserves further investigation in the future.

**Table 2:** The diagnostic potential of m^6^A and PCD in Atherosclerosis

Technique	M^6^A associated molecules	Expression	M^6^A levels	Model	Ref
RNA-seq	METTL3	Increased	Increased	PBMC derived macrophage RNA in patients with coronary artery disease (CAD)	(Zhou et al. 2022a)
RNA-Seq and meRIP-Seq	METTL3	Increased	Increased	human coronary artery smooth muscle cells (HCASMCs)	(Guo et al. 2020)

### Therapeutic potential

METTL3 is one of the major m^6^A modified proteins most commonly reported to be associated with the development of atherosclerosis. Upregulated METTL3 increases the m^6^A level of NLRP3 mRNA, leading to the release of pro-inflammatory cytokines from macrophages. Moreover, miR-1208 can inhibit the expression of NLRP3 and lead to decreased release of inflammatory cytokines after targeted binding to METTL3, suggesting that miR-1208 and its downstream gene METTL3 are potential targets for the prevention and treatment of atherosclerosis (Zhou et al. 2022a, b). Vitamin D3 is a multifunctional fat-soluble hormone that is essential in atherosclerosis (Lu et al. 2020; Bobryshev 2010). Recent studies have shown that vitamin D3 downregulates METTL3 by inhibiting AMPK activation, thereby inhibiting m^6^A modification and apoptosis of vascular endothelial cells (Zhu et al. 2022). This finding adds to the understanding of the m^6^A driving mechanism in vascular endothelial injury and highlights the significance of METTL3 in the intervention of atherosclerosis. In addition, inhibition of FTO is closely related to anti-atherosclerosis by regulating the PCD pathway. In Luo et al.‘s study, nanoparticle-mediated delivery of FTO-siRNA or administration of the FTO inhibitor entacapone inhibited lipopolysaccharide-induced macrophage pyroptosis (Luo et al. 2021). Mechanistically, ablation of FTO inhibits the NLRP3 inflammasome in macrophages through the forkhead box protein O1/nuclear factor kappa-B (FOXO1/ NF-κB) signalling pathway. Accordingly, inhibition of FTO is expected to be used in the treatment of atherosclerosis. It is well known that impaired blood flow and associated oscillatory stress (OS) play a central role in the development of atherosclerosis. Chien et al. investigated the role of m^6^A methylation in mechanical transduction of endothelial cells (ECs) (Chien et al. 2021). They have determined that OS causes hypermethylation of m^6^A RNA, in which the m^6^A methyltransferase METTL3 is the center of response to hemodynamic and atherogenic stimuli in ECs. Further RNA sequencing and m^6^A enhanced cross-linking and immunoprecipitation (eCLIP) experiments showed that NLRP3 is a hemodynamically related downstream target of METTL3-mediated hypermethylation, recognized by the m^6^A reader protein YTHDF1. In the *vivo* atherosclerosis model, repeated administration of METTL3 shRNA prevented the atherogenic process and the upregulation of NLRP3. Together, these results support the clinical therapeutic potential of the m^6^A-PCD axis in the development and progression of atherosclerosis (Table 3). In the near future, specific m^6^A- modified targeted drugs may be developed and used in preclinical and clinically validated treatment of atherosclerosis.

**Table 3:** The targeted therapy of m^6^A and PCD in atherosclerosis

Targeted therapy	M^6^A associated molecules	Expression	Molecular mechanisms	Model	Ref
EVs derived from human umbilical cord mesenchymal stem cells	METTL3	Decreased	EVs decreased the m^6^A level of NLRP3 mRNA following miR-1208 targeted binding to METTL3, resulting in decreased inflammatory factor release	THP-1 cells	(Zhou et al. 2022b)
Vitamin D3	METTL3	Decreased	Decreased METTL3 expression by inhibiting AMPK activation, thereby inhibiting m^6^A modification and apoptosis of vascular endothelial cells	Human cytomegalovirus infection induced vascular endothelial cells apoptosis	(Zhu et al. 2022)
Nanoparticle-mediated FTO-siRNA	FTO	Decreased	Ablation of FTO could inhibit NLRP3 inflammasome through FoxO1/NF-κB signaling	LPS-induced macrophages	(Luo et al. 2021)
Entacapone	FTO	Decreased	Ablation of FTO could inhibit NLRP3 inflammasome through FoxO1/NF-κB signaling	LPS-induced macrophages	(Luo et al. 2021)

## Concluding remarks and future perspectives

A large number of studies have confirmed that m^6^A modification is an important target for the treatment of atherosclerosis and that improving m^6^A level can effectively reduce the progression of atherosclerosis (Yang et al. 2023; Zhang et al. 2023a, b; Yu et al. 2022). A High-fat diet is a major cause of atherosclerosis. Evidence suggests that maternal consumption of a high-fat diet increases m^6^A modification in offspring, which can lead to elevated triglycerides and altered pro-inflammatory profiles in adult offspring (Izquierdo et al. 2021). These findings have prompted researchers to investigate the role of m^6^A in atherosclerosis.

In this review, we summarize the potential function of m^6^A methylation in PCD, particularly in the context of atherosclerosis. In addition, we highlight the promise of diagnostics and therapies based on interference with m^6^A in PCD, which could have a transformative impact on clinical medicine. However, the current understanding of the relationship between the m^6^A-PCD axis and atherosclerosis is only the tip of the iceberg. Objectively speaking, some of the functions of m^6^A in PCD are inferred from the known studies involving this mRNA modification. Despite recent progress, many challenging problems remain in fully understanding the specific mechanisms of m^6^A and PCD in the development of atherosclerosis (see Outstanding questions). Further research is urgently needed to explore in depth.

Firstly, a plethora of unexplored m^6^A regulators in programmed cell death (PCD) awaits discovery, including YTHDF1, YTHDF1, YTHDC1, YTHDC2, and others. Secondly, the investigation into m^6^A-mediated PCD within atherosclerosis-associated cells, such as macrophages and vascular smooth muscle cells (VSMCs), remains limited, potentially harboring undiscovered effects. Thirdly, m^6^A regulatory factors encompass inhibitors of the METTL3-METTL14 complex (3-deazaadenosine, eltrombopag) (Feng et al. 2022; Yankova et al. 2021; Buker et al. 2020), and FTO inhibitors (FB23-2, entacarone) (Huang et al. 2019; Peng et al. 2019). Whether these compounds precisely modulate atherosclerosis by controlling PCD pathways remains elusive. Fourthly, the crosstalk between different PCDs with a particular m^6^A modification enzyme still needs to be further investigated. Lastly, the interaction between different epigenetic modifications on PCD in atherosclerosis has not been studied.

We believe that the development of m^6^A-PCD axis-related targeted therapies in atherosclerosis will be a focus in the near future. Currently, targeting m^6^A via the writing enzyme METTL3 or the erasing enzyme FTO appears to be the most promising new therapy in atherosclerosis. With existing technological advances, targeted therapy for atherosclerosis based on the m^6^A-PCD axis will provide more options for cardiologists. The rewards would be enormous if we could use the m^6^A-PCD axis to predict and intervene in the progression of atherosclerosis.

## Data Availability

No datasets were generated or analysed during the current study.
